# Evaluating the Antitumor Effect of Root Extract of Salvadora persica in Hepatocarcinoma Through the Induction of Apoptosis via an Intrinsic Pathway: An In Vitro Study

**DOI:** 10.7759/cureus.93847

**Published:** 2025-10-04

**Authors:** Fouzia Latif, Kong Qun, Mingjing Lu

**Affiliations:** 1 Faculty of Medicine, Qilu Institute of Technology, Jinan, CHN

**Keywords:** antitumor, apoptotic, aqueous extract, cytotoxic, hepatocarcinoma, salvadora persica

## Abstract

Background: Hepatocarcinoma (HCC) is the primary form of liver cancer, which is a highly prevalent cancer associated with an increased rate of mortality worldwide. The plant *Salvadora persica* (miswak) has been reported for its cytotoxic effect in various cancer cell lines. The purpose of the current research study was to assess the cytotoxicity and apoptosis-inducing capability of *Salvadora persica* aqueous extract from the root part (miswak sticks) on human HCC HepG2 cells and to evaluate its safety in human normal hepatic THLE-2 cells in a dose-dependent manner.

Materials and methods: The* *preparation of aqueous extract from the root part of *Salvadora persica *was carried out using a maceration extraction process. The effect on cytotoxicity and cellular proliferation in HepG2 cells and THLE-2 cells at 10, 50, 100, 150, and 200 µg/mL concentrations after 24, 48, and 72 hours was determined using the MTT (3-(4,5-dimethylthiazol-2-yl)-2,5-diphenyltetrazolium bromide) assay. In further experiments, IC50 (24 hours) was 65.8 µg/mL, and increasing concentrations were employed: 115 µg/mL (twofold) and 165 µg/mL (threefold). The apoptosis at the quantitative level was evaluated using a flow cytometry assay by the Annexin V-FITC/PI apoptosis detection kit in HepG2 cells and THLE-2 cells. The expression levels of the proapoptotic proteins Annexin V and p53 in the HepG2 cell line were assessed using an immunocytochemistry assay. The mRNA levels of apoptosis markers, caspase 9, caspase 3, cytochrome c, BAX, and p53, and the antiapoptotic marker, Bcl-2, in the HepG2 cell line were determined using the RT-qPCR (real-time, quantitative-polymerase chain reaction) assay.

Results: *Salvadora persica *aqueous extract demonstrated time-dependent and dose-dependent growth inhibitory effect and cytotoxicity in the HepG2 cell line in the MTT test. The IC50 values were 65.8 µg/mL at 24 hours, 51.4 µg/mL at 48 hours, and 30.3 µg/mL at 72 hours. In contrast, a non-toxic effect was observed in the THLE-2 cell line. The flow cytometry results indicated a significantly enhanced apoptotic effect, characterized by increased early apoptosis, late apoptosis, and cell death in HepG2 cells. In contrast, the normal THLE-2 cell line showed insignificant apoptotic cell numbers. In the immunostaining assay, a pronounced elevation in the expression levels of proapoptotic proteins, including p53 and Annexin V, was observed. The RT-qPCR assay revealed an increase in the fold change of caspase 9, caspase 3, cytochrome c, BAX, and p53 mRNA levels, along with a decrease in Bcl-2 mRNA levels. *Salvadora persica a*queous extract induced apoptosis via the intrinsic pathway in the HepG2 cell line. *Salvadora persica *aqueous extract exhibited the most significant apoptotic effect in HepG2 cells at high concentrations.

Conclusions: In the present study, *Salvadora persica *aqueous extract demonstrated dose-dependent antitumor potential in the HCC cell line HepG2 through the induction of intrinsic apoptosis and showed non-toxic activity in the normal hepatic THLE-2 cell line, emphasizing its therapeutic efficacy in HCC treatment.

## Introduction

Hepatocarcinoma (HCC) is the primary form of liver cancer, which is rated as the sixth most prevalent cancer and ranked the third leading cancer with increased global mortality [1]. HCC risk factors are hepatitis B, hepatitis C, fibrosis, cirrhosis, non-alcoholic fatty liver, and steatohepatitis. The prevalence of HCC is two to four times higher in males than in females [2]. The apoptosis process is defined as programmed cell death that is preceded by two distinct pathways: extrinsic apoptosis and intrinsic apoptosis [3]. Cancer cells evade apoptosis due to an imbalance in the regulation of antiapoptotic and proapoptotic proteins, resulting in chemotherapy-resistant cancer cells [4]. Tumor protein p53 acts as a suppressor of tumor cells and is genetically involved in both extrinsic and intrinsic apoptosis; however, it has a particularly strong influence on the mitochondrial intrinsic apoptosis process [5]. During the apoptosis process, phosphatidylserine is translocated from the outer cell membrane to the inner side of the cell, serving as a marker for the diagnosis of apoptosis [6]. The mutations cause inactivation of the tumor suppressor p53, resulting in loss of control over cancerous cell growth and division, as well as resistance to anticancer drugs [5]. The effectiveness of HCC drugs has increased over time, largely due to the advent of targeted therapies and immunotherapies [7]. The present research interest has shifted to plant extracts for HCC treatment due to their antitumor activity, which involves exerting cytotoxicity, blocking different cell phases, and inducing apoptosis effects, while offering fewer adverse effects with better cost-effectiveness [8]. It is evident that herbal remedies offer therapeutic benefits and are effective in treating HCC by inducing both intrinsic and extrinsic apoptosis, thereby limiting the growth of cancer cells [9]. *Salvadora persica* (*S. persica*), also known as miswak, belonging to the family Salvadoraceae, is well recognized for its diverse range of pharmacological activities, including antioxidant, anticancer, antimicrobial, antiulcer, anti-inflammatory, diuretic, antidiabetic, and analgesic properties [10,11]. From the root part of *S. persica*, miswak sticks for cleaning teeth are prepared, containing important phytoconstituents such as glycosides, alkaloids, tannins, flavonoids, saponins, phenolic acids, calcium, and ascorbic acid [12,13]. To the best of our knowledge, this is the first study to evaluate the antitumor activity of the root extract of *S. persica* and its underlying molecular mechanism in the HCC HepG2 cell line, as well as to assess its safety in THLE-2 cells. Hence, this study aims to investigate the in vitro cytotoxic and apoptotic effects of *S. persica*-Aq extract in HepG2 and THLE-2 cell lines, with a focus on exploring its apoptotic mechanism. This study employed increasing concentrations of *S. persica*-Aq extract to assess the safety in the THLE-2 cell line by observing the selectivity of *S. persica*-Aq extract in human hepatocellular carcinoma cells, HepG2, compared with human normal liver cells, THLE-2.

## Materials and methods

Reagents and chemicals

MTT (3-(4,5-dimethylthiazol-2-yl)-2,5-diphenyltetrazolium bromide) dye, Dulbecco’s Modified Eagle’s Medium-high glucose (DMEM-HG), fetal bovine serum (FBS), 4,6-diamidino-2-phenyl indole (DAPI) dye, dimethyl sulfoxide (DMSO), phosphate buffer saline (PBS), bovine serum albumin, TRIS buffer saline with Tween 20 (TBS-T), Annexin-V-FITC/PI apoptosis detection kit, penicillin, and streptomycin were purchased from Sigma-Aldrich, USA. Rabbit polyclonal primary antibody of p53 and Annexin V, secondary antibody donkey anti-rabbit IgG-FITC conjugate, MMLV reverse transcriptase commercial kit, and SYBR Green Master Mix were purchased from Invitrogen, Thermo Fisher Scientific, USA.

Plant *S. persica* extract

The miswak sticks, prepared from the root part of the plant *S. persica*, were bought from a well-known herbal supplier, Sichuan Wekeqi Biotechnology Company in Chengdu, China. Professor Nan Xu of the Qilu Institute of Technology, Jinan, Shandong, China, verified the plant part. In the herbarium of the Medical Department, the voucher number 3856 was deposited. Miswak sticks were crushed into powder form. One hundred grams of miswak sticks powder was macerated in 1 L of aqueous solvent at room temperature for 72 hours. Then, it was filtered using Whatman filter paper (grade 4), and the obtained filtrate was evaporated under reduced pressure at 40°C using a rotary evaporator. Later, this was air-dried, and a product yield of 19 g was obtained. The final form of *S. persica*-Aq extract was stored at -20°C and utilized for subsequent experiments.

HepG2 cells and THLE-2 cells culturing

The human HCC HepG2 cells and the human normal liver THLE-2 cell line were procured by the Cell Bank of Qilu Institute of Technology, Jinan, China. Cell culturing of HepG2 and THLE-2 cell lines was carried out in 10% FBS, 100 U streptomycin, and 100 U penicillin, containing DMEM-HG medium at 37°C, 5% CO2, and 95% humidity. The experiments were performed when the cultured HepG2 and THLE-2 cells attained 80-90% confluence.

MTT assay

The cytotoxic effect of *S. persica*-Aq extract on HepG2 and THLE-2 cell lines (5 × 10³ cells/well) in a 96-well plate was assessed using the widely used MTT assay, as reported earlier [14]. Different dilutions of *S. persica*-Aq extract, ranging from 10 to 200 µg/mL, prepared in DMSO, were applied to HepG2 and THLE-2 cell lines separately at 37°C. Then, 10 µL of the yellow MTT dye was administered to the wells, and the plate was incubated in the dark for three hours. The viable, or live, cells reduced the MTT yellow dye into purple-colored formazan crystals. Then, formazan crystals were dissolved by the addition of DMSO. After shaking it gently for up to 10 minutes at 25°C, the absorbance was measured spectrophotometrically (Thermo Fisher Scientific, MA, USA) at 570 nm.

Apoptosis determination using Annexin V-FITC/PI detection kit by flow cytometry assay

A flow cytometry assay using an apoptosis detection kit, Annexin V-FITC/PI, was performed on *S. persica*-Aq extract-treated HepG2 and THLE-2 cell lines to determine the different phases of apoptosis, as previously reported [15]. Annexin V detects exposed phosphatidylserine in the outer cell membrane of compromised, dead cells, while propidium iodide detects compromised plasma membranes. It intercalates into damaged DNA and emits fluorescence while remaining impermeable to healthy cell DNA. Due to the deteriorated integrity of the plasma membrane, late apoptotic cells are differentiated from early apoptotic cells by staining with propidium iodide. In a six-well plate, *S. persica*-Aq extract at an IC50 value of 65.8 µg/mL and its increasing doses of 115 µg/mL (twofold) and 165 µg/mL (threefold) were administered in wells (3 × 105 cells/well) containing HepG2 cells and THLE-2 cells, separately, and incubated at 37°C in a temperature-controlled incubator for 24 hours. After removing the culture medium, the HepG2 and THLE-2 cells in the wells were washed three times with PBS. The cells were then detached by adding trypsin for three to five minutes, and the addition of medium containing FBS terminated the reaction. Then, the cell suspension was collected and centrifuged at 1000 rpm for five minutes at 4°C. After removing the supernatant, the cells were resuspended in 100 µL of cold annexin V binding buffer. Later, 5 µL of Annexin V-FITC was added, and the mixture was gently mixed. This was followed by a 1.5-minute incubation of the cells at room temperature in the dark. Then, propidium iodide at 5 µL was added to the mixture and incubated for five minutes in darkness, followed by cell resuspension in binding buffer (100 µL). After that, quantitative apoptosis was determined by flow cytometry (Agilent Novocyte, CA, USA) using FlowJo software version 10.0 (FlowJo, LLC, OR, USA).

Immunocytochemistry assay

The expression levels of apoptotic proteins, p53 and Annexin V, were measured in *S. persica*-Aq extract-treated HepG2 cell lines after 24 hours using the immunocytochemistry assay according to a previously described method [16]. The *S. persica*-Aq extract at an IC50 value of 65.8 µg/mL and its increasing doses of 115 µg/mL (twofold) and 165 µg/mL (threefold) were administered in a six-well plate (3 × 105 cells/well) that contained HepG2 cells and incubated at 37°C for 24 hours. Later, the medium in the wells was removed, and the cells were washed with TBS-T (1X) five times. The 200 µL of paraformaldehyde (4%) was administered to cells at 25°C for 30 minutes, and the cells were washed with TBS-T (1X) five times. After that, 200 µL of blocking solution (5% bovine serum albumin) was applied to the wells for 25 minutes, and then the wells were washed five times using TBS-T (1X). Then, rabbit polyclonal primary antibodies against p53 and Annexin V were added to wells containing the HepG2 cell line, and the cells were incubated at 37°C for 1.5 hours. Following incubation, the antibodies were removed, and the wells were washed five times with TBS-T (1X). Later, a secondary antibody, FITC-conjugated donkey anti-rabbit, was applied to the cells, incubated at 37°C for 1.5 hours, and then removed and washed with TBS-T (1X) five times. Then, DAPI at 1 µg/mL in PBS was applied to the cells and incubated for 15 minutes at 25°C. The cells were then washed five times using TBS-T (1X). Later, stained HepG2 cells were observed under the Floid® Cell Imaging Station (Thermo Fisher Scientific, MA, USA). HepG2 cells were stained green, while nuclei were stained blue.

Genomic profile using RT-qPCR assay

The apoptotic markers, caspase 3, caspase 9, cytochrome c, BAX, and p53 mRNA levels, and the anti-apoptotic marker Bcl-2 mRNA levels in HepG2 cells treated with increasing concentrations of *S. persica*-Aq extract after 24 hours of incubation was assessed via RT-qPCR. The human primer set for the RT-qPCR assay was designed using the PrimerQuest tool (Integrated DNA Technologies, IA, USA) and validated with Primer-Blast (National Center for Biotechnology Information, MD, USA). These primers were used to obtain quantification by the thermocycler, with HPRT serving as the housekeeping gene (Table 1). The primers were manufactured by ShineGene Biotechnology (China). The Trizol reagent was used to extract total RNA from HepG2 cells, and its concentration was determined using the NanoPhotometer spectrophotometer (Implen, Germany). The isolated total RNA (2 µg) was then used to prepare cDNA using a commercial MMLV reverse transcriptase kit (Invitrogen, Thermo Fisher Scientific, MA, USA), following the manufacturer's protocol. Gene mRNA levels were measured using a qTower3 thermocycler (Analytic Jena AG, Germany). The amplification of DNA using SYBR Green PCR Master Mix (Invitrogen, Thermo Fisher Scientific, MA, USA) was performed according to the manufacturer's described cycling conditions, with HPRT used as an internal control. The gene's relative mRNA expression level was expressed as a fold change, determined using the ΔΔCt method.

**Table 1:** Primer sequence of proapoptotic markers and antiapoptotic marker Forward (F) and reverse (R) sequences of human primer for RT-qPCR assay. RT-qPCR: reverse transcription quantitative polymerase chain reaction

No.	Primer	Sequence	Annealing temperature
	Human		
1	p53	F: GGCCCACTTCACCGTACTAA	63
		R: GTGGTTTCAAGGCCAGATGT	
2	Bax	F: GCCTCCTCTCCTACTTTGG	68.7
		R: CCTCCCAGAAAAATGCCATA	
3	Cytochrome c	F: ACGGCAGGCAAGCACTAGACTA	3
		R: GGCCATCAGCGTATAATCTCCTA	
4	Caspase 3	F: GAA GAA CTT AGG CAT CTG TG	52
		R: GCA TAG GGA CAA ATC AGA AG	
5	Caspase 9	F: GGGTGACGACCTTCGAACAAGT	60
		R: AACACGTACAACGTTGCACCATT	
6	BCL-2	F: ATCGTGTACCGGATGACTGAGT	62
		R: GTCATGAGCAATCCAACAAAGC	
7	HPRT	F: GAACGTCTTGCTCGAGATGTG	57
		R: CCAGCAGGTCAGCAAAGAATT	

Statistical analysis

The data from experiments were expressed as mean ± SD. A one-way ANOVA with the Bonferroni test was applied to the statistical analysis of the data. GraphPad Prism version 10.4.1 (GraphPad Software, Inc., MA, USA) was used for statistical data analysis. A p-value of less than 0.05 determined statistical significance.

## Results

Effect of *S. persica* extract on cell viability in MTT assay

**Figure 1:**
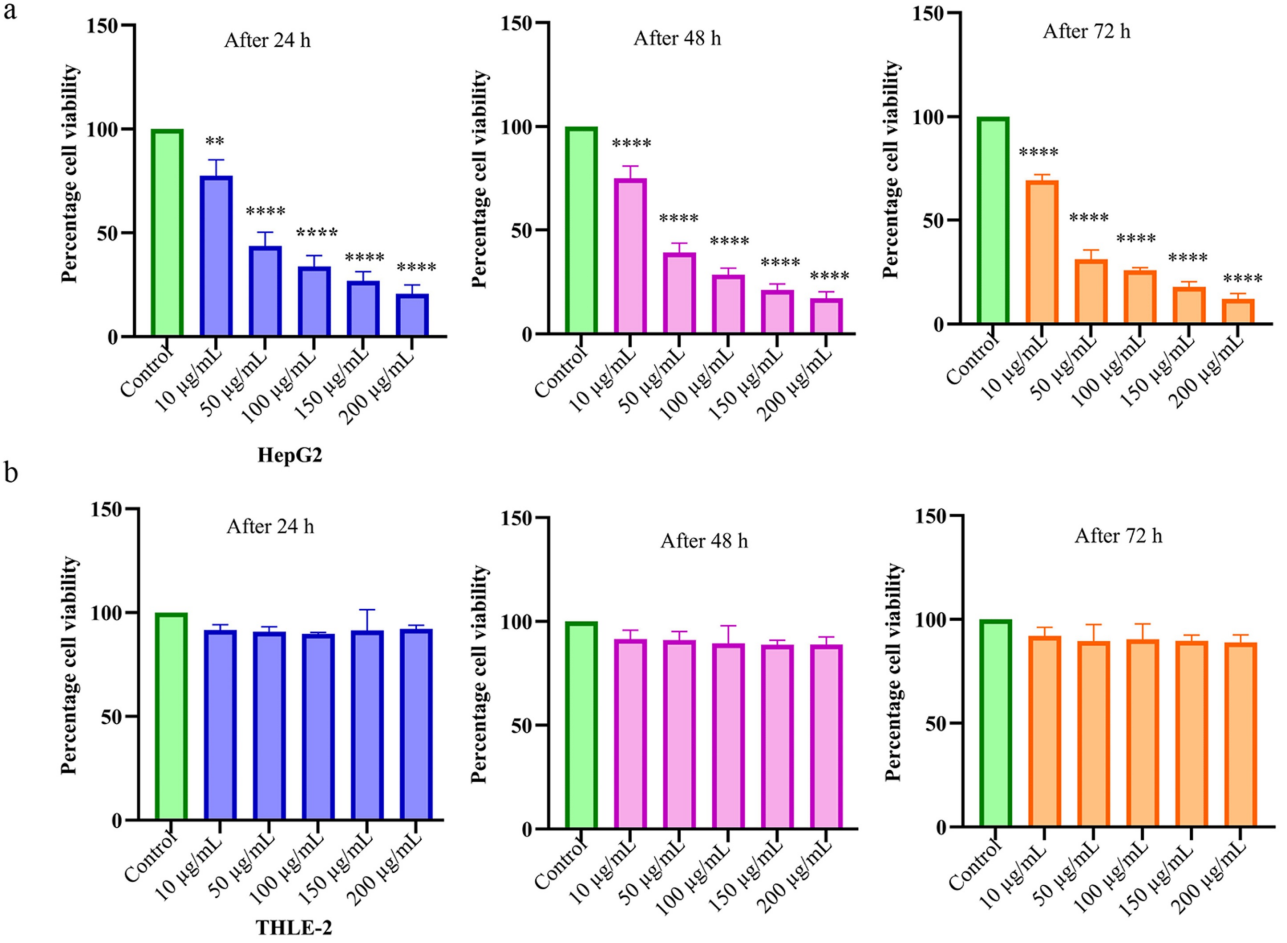
Effect of S. persica extract on viability in HepG2 and THLE-2 cells Percentage cell viability in (a) HepG2 and (b) THLE-2 cell lines, treated at 10-200 µg/mL concentrations of *S. persica*-Aq extract after 24, 48, and 72 hours. The data are expressed as mean ± SD of triplicate experiments. ** p < 0.01 and ** p < 0.0001 were statistically significant compared to the control group. SD: standard deviation, *S. persica*: *Salvadora persica*

A widely used colorimetric MTT assay was employed for determining the cytotoxic potential of *S. persica*-Aq extract at increasing concentrations in HCC HepG2 cells and normal hepatic THLE-2 cells. *S. persica*-Aq extract demonstrated a significant decrease in cell viability in HepG2. The IC50 values were 65.8 µg/mL (24 hours), 51.4 µg/mL (48 hours), and 30.3 µg/mL (72 hours). In contrast, the *S. persica*-Aq extract showed negligible cytotoxicity in THLE-2 cells. These results indicate that *S. persica*-Aq extract exerted a cytotoxic effect in the cancerous HepG2 cell line in a time-dependent and concentration-dependent manner and exhibited a harmless effect in the normal THLE-2 cell line (Figure 1).

Impact of *S. persica* extract on apoptosis by flow cytometry

**Figure 2:**
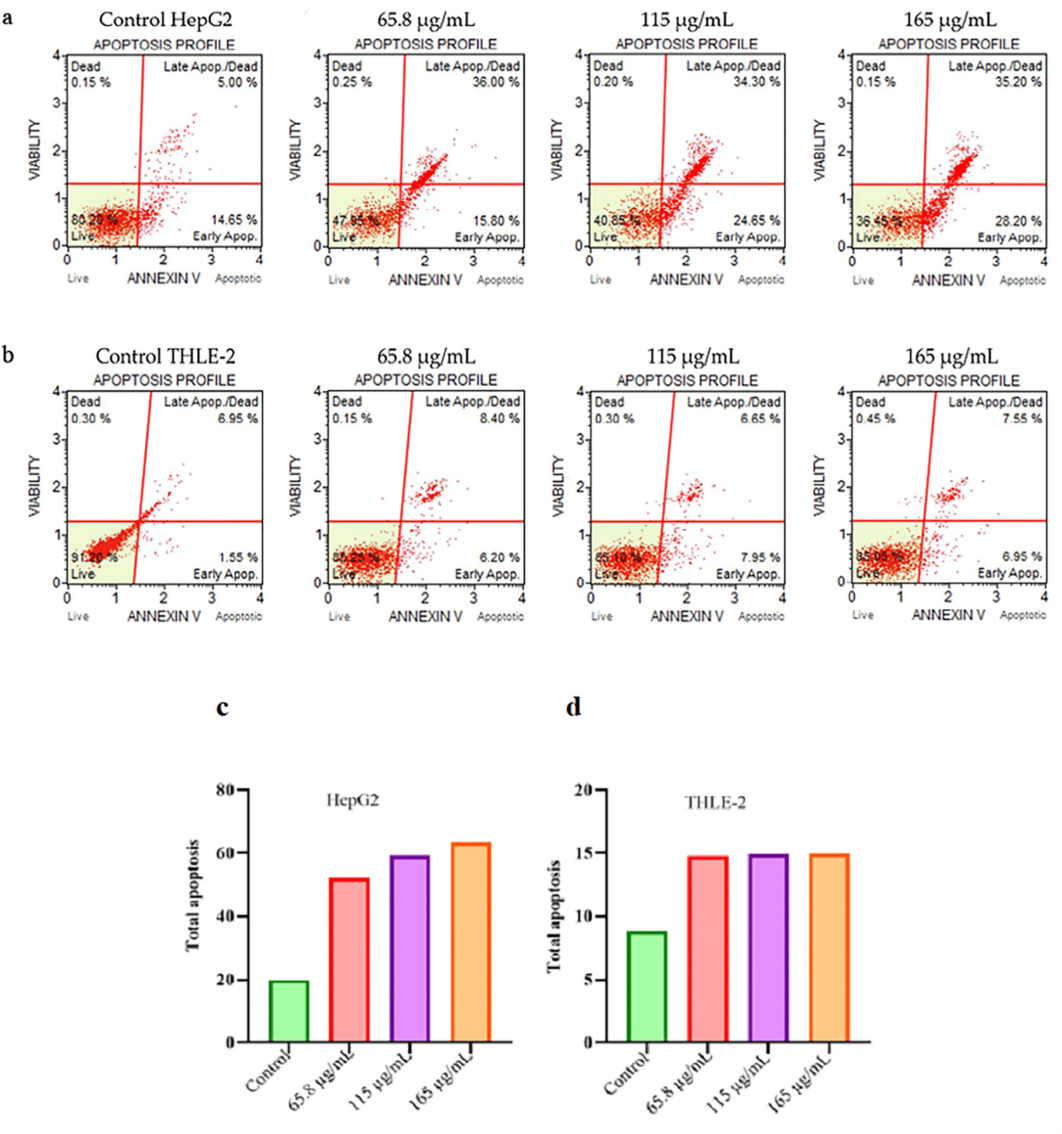
S. persica extract apoptosis effect in HepG2 cells and THLE-2 cells after staining with Annexin V FITC/PI apoptosis detection kit At increasing concentrations of *S. persica*-Aq extract, the treated HepG2 cell line and the treated THLE-2 cell line were evaluated for apoptosis in different phases after 24 hours using flow cytometry. The total apoptosis in (c) HepG2 and (d) THLE-2 cell lines was also assessed. *S. persica*: *Salvadora persica*

The effect of *S. persica*-Aq extract at increasing concentrations on apoptosis in different phases of the HepG2 cell line and THLE-2 cell line was measured using an Annexin V-FITC/PI detection kit for apoptosis after 24 hours by flow cytometry. The study findings revealed that *S. persica*-Aq extract induced significant apoptosis in the HepG2 cell line in a concentration-dependent manner, compared to the control group. At the same time, *S. persica*-Aq extract showed a non-noticeable apoptotic effect in the THLE-2 cell line. Total apoptosis was calculated by summing the number of dead cells, late apoptotic cells, and early apoptotic cells in the HepG2 and THLE-2 cell lines. *S. persica*-Aq extract in cancerous HepG2 cells at different concentrations of 65.8, 115, and 165 µg/mL induced total apoptosis of 52.05%, 59.15%, and 63.55%, respectively, as compared to 19.8% in the control group. While in normal THLE-2 cells, *S. persica*-Aq extract at concentrations of 65.8, 115, and 165 µg/mL induced total apoptosis of 14.75%, 14.9%, and 14.95%, respectively, compared to 8.8% in the control group. These findings suggest that *S. persica*-Aq extract is more selective to cancerous HepG2 cells than normal THLE-2 cells (Figure 2a-2d, Table 2).

**Table 2:** Quantitative apoptosis analysis in HepG2 and THLE-2 cell lines The apoptosis at the quantitative level was determined in the HepG2 cell line and the THLE-2 cell line after treatment with *S. persica*-Aq extract at various concentrations (µg/mL) for 24 hours using the Annexin V-FITC/PI apoptosis kit. *S. persica*: *Salvadora persica*

	HepG2 cells percentage (%)				THLE-2 cells percentage (%)				
Groups	Dead	Late apoptosis	Early apoptosis	Live	Dead	Late apoptosis	Early apoptosis	Live	
										Control	0.15	5	14.65	80.2	0.3	6.95	1.55	91.2	
Extract 65.8 µg/mL	0.25	36	15.8	47.95	0.15	8.4	6.2	85.25	
Extract 115 µg/mL	0.2	34.3	24.65	40.85	0.3	6.65	7.95	85.1	
Extract 165 µg/mL	0.15	35.2	28.2	36.45	0.45	7.55	6.95	85.05	

Influence of *S. persica* extract on proapoptotic proteins in an immunocytochemistry assay

**Figure 3:**
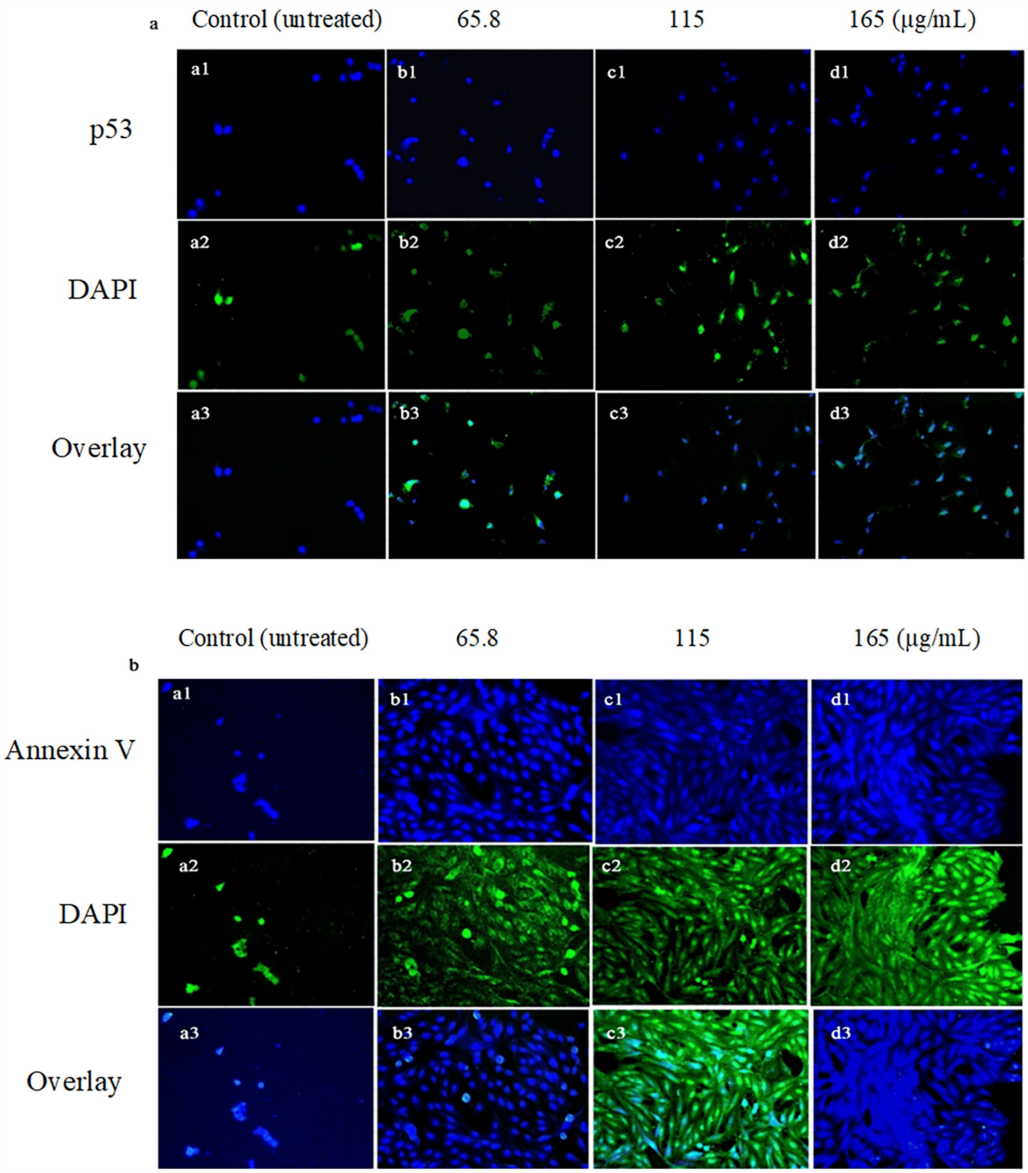
Impact of S. persica extract on the expression level of proapoptotic proteins in HepG2 cells The HepG2 cells were treated with *S. persica*-Aq extract at increasing concentrations for 24 hours, and expression levels of proapoptotic proteins, (a) p53 and (b) Annexin V, were measured by immunocytochemistry assay. *S. persica*: *Salvadora persica*

The impact of *S. persica*-Aq extract at increasing concentrations on p53 levels and Annexin V levels in HepG2, an HCC cell line, over 24 hours was evaluated using an immunocytochemistry assay. A significant increase in p53 and Annexin V expression levels was shown in the extract-treated HepG2 cell groups compared to the untreated control group. *S. persica*-Aq extract showed a concentration-dependent apoptotic response in HepG2 cells (Figure 3a-3b).

Genomic profile analysis in *S. persica* extract-treated HepG2 cells by real-time qPCR assay

**Figure 4:**
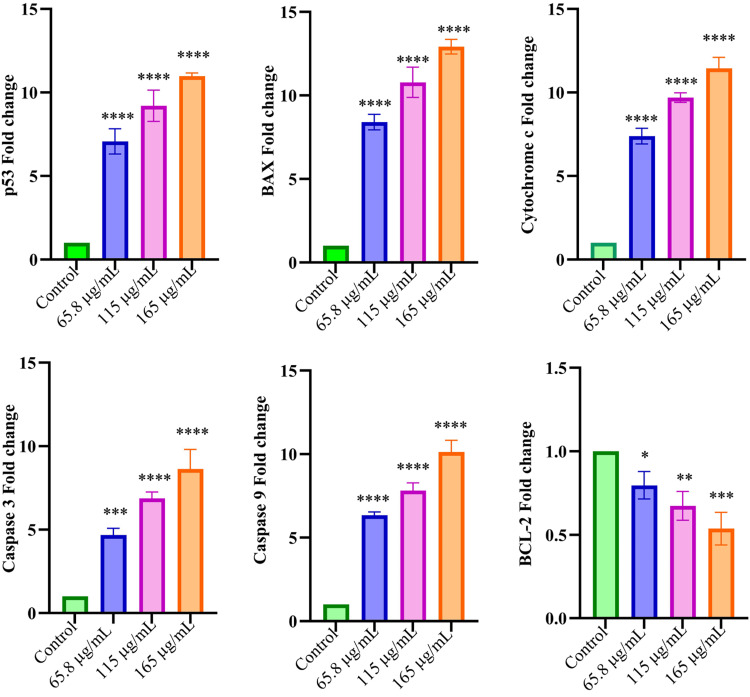
S. persica extract effect on targeted proapoptotic markers and antiapoptotic markers in HepG2 cells using RT-qPCR assay The effect of *S. persica*-Aq extract at increasing concentrations on the mRNA level of proapoptotic and antiapoptotic markers (expressed in fold change) in the HCC HepG2 cell line was determined by RT-qPCR assay. The statistical analysis, using one-way ANOVA with the Bonferroni test, was applied to the data presented as mean ± SD. p ≤ 0.1; *p ≤ 0.01; ***p ≤ 0.001; and ****p ≤ 0.0001 were statistically significant in comparison with the control. *S. persica*: *Salvadora persica, *RT-qPCR*: *reverse transcription quantitative polymerase chain reaction, HCC: hepatocarcinoma, SD: standard deviation, ANOVA: analysis of variance

The increasing concentrations of *S. persica*-Aq extract were applied to HCC HepG2 cells, and after 24-hour incubation, the expressions of proapoptotic and antiapoptotic markers were measured using RT-qPCR. The mRNA expression levels of the markers were expressed as fold changes. A significantly upregulated level of proapoptotic markers and a downregulated level of antiapoptotic markers were observed. At *S. persica* extract at various concentrations (µg/mL): 65.8, 115, and 165. The increase in fold change of apoptotic markers' mRNA level was as follows: p53 gene 7.0, 9.3, and 10.8; BAX gene 8.3, 10.8, and 13.1; cytochrome c gene 7.4, 9.7, and 11.5; caspase 3 gene 4.8, 6.7, and 8.7; and caspase 9 gene 6.4, 7.7, and 10, respectively. While the antiapoptotic Bcl2 gene fold decrease in mRNA level was 0.71, 0.6, and 0.5 at 65.8, 115, and 165 concentrations (µg/mL), respectively (Figure 4).

## Discussion

The current challenges in chemotherapy for highly prevalent HCC worldwide have urged the need to explore herbal products that provide therapeutic benefits with fewer side effects in HCC by triggering cytotoxicity and apoptosis in cancer cells [17]. Plant *S. persica* is well recognized for its multiple pharmacological activities in the treatment of various ailments and has been reported for its cytotoxic potential against various cancer cell lines [18]. The present research study aimed to initially screen the in vitro anticancer potential of *S. persica*-Aq extract on HCC HepG2 cells, with a focus on exploring its apoptosis mechanism and assessing its safety in normal hepatic cells THLE-2 in a dose-dependent manner. This research study employed increased concentrations of extract to assess the selective cell cytotoxicity between HepG2 cancerous cells and THLE-2 normal healthy cells. In the current research work, the *S. persica*-Aq extract demonstrated remarkable cytotoxic activity in HepG2 cells, resulting in cellular growth inhibition in a time- and concentration-dependent manner, as determined by the MTT assay. The extract IC50 concentrations (µg/mL) were 65.8 (24 hours), 51.4 (48 hours), and 30.3 (72 hours). While *S. persica*-Aq extract showed non-noticeable cytotoxicity in normal hepatic THLE-2 cells, indicating that the extract is selective towards HCC and HepG2 cells and is safe in normal THLE-2 cells (Figure 1). These cytotoxic findings of *S. persica*-Aq extract are consistent with a previous study that reported the cytotoxicity of *S. persica* aqueous extract in cancerous cells, specifically in PE/CA-PJ15 (oral squamous cancer) and MCF-7 (breast cancer) cell lines [19,20]. In this study, the influence of *S. persica*-Aq extract on apoptosis at increasing concentrations in the HepG2 cell line was evaluated using a flow cytometry-based Annexin V-FITC/PI detection kit. A pronounced apoptosis was observed, accompanied by a significant increase in dead cells, as well as late and early apoptotic cells, compared to the control.

In contrast, insignificant apoptosis was shown in THLE-2 normal cells (Figure 2a-2d, Table 2). These results also align with the cytotoxicity results of the MTT assay, in which the extract demonstrated significant cytotoxicity in the HepG2 cell line and negligible cytotoxicity in the THLE-2 cell line. The *S. persica*-Aq extract, which exhibits cytotoxic potential, may be related to its apoptotic effect. In our study, *S. persica*-Aq extract demonstrated a concentration-dependent, significant increase in the expression of p53 protein and Annexin-V protein levels, as determined by the immunostaining assay (Figure 3a-3b). These findings of increasing proapoptotic protein expression are also consistent with findings of the flow cytometry assay. The p53 functions as a transcription mediator that activates proapoptotic BAX gene activation, which causes destabilization of the mitochondrial membrane, and the balance between Bcl2/BAX is disturbed, resulting in mitochondrial cytochrome c secretion to the cytosol, which forms the apoptosome with caspase 9, which later activates caspase 3 to execute apoptosis [21,22]. At increasing concentrations, the impact of *S. persica*-Aq extract on the mRNA expression, as measured by the fold change, of apoptotic and antiapoptotic markers was determined in the HepG2 cell line using an RT-qPCR assay. The *S. persica*-Aq extract significantly upregulated the mRNA levels of apoptotic genes, including p53, BAX, caspases 3 and 9, and cytochrome c, and induced apoptosis through the intrinsic pathway. The *S. persica*-Aq extract showed downregulation of the Bcl-2 mRNA level, an antiapoptotic marker (Figure 4). The *S. persica*-Aq extract significantly increased apoptotic potential in response to increased extract concentration. These apoptosis results are also in line with current flow cytometry and immunocytochemical assay results, which show that *S. persica*-Aq extract exhibits apoptotic activity in a dose-dependent manner. The present research study utilized the increasing concentrations of extract to assess its selective cell cytotoxicity between normal and cancerous cells. Our research indicated that *S. persica*-Aq extract exhibited cytotoxic activity and induced apoptosis through the intrinsic pathway in HepG2 cells.

In contrast, it showed a harmless effect with negligible cytotoxicity and apoptosis in THLE-2 cells. The study also confirms that *S. persica*-Aq extract at higher concentrations is selective for cancerous cells, HepG2, and non-toxic and safe in the normal hepatic THLE-2 cell line. This study provided preliminary evidence for the therapeutic potential of *S. persica* aqueous extract in the HCC cell line.

Limitations of the study

The current study investigated the in vitro cytotoxic and apoptotic effects of *S. persica*-Aq extract through the intrinsic pathway in HCC HepG2 cells and its safe effects in the normal hepatic THLE-2 cell line. But this study still has a few limitations that need to be addressed in the future. In vivo research using *S. persica* extract is required to confirm its efficacy in the HCC-induced model and assess its safety in the normal liver. In vivo, an apoptotic study using the *S. persica* extract is necessary to explore the intrinsic and extrinsic apoptotic pathways in detail, targeting downstream apoptosis mediators, and to gain a clear understanding of the therapeutic efficacy of *S. persica* extract for HCC treatment.

## Conclusions

In our study, *S. persica* Aq extract showed anticancer activity in the HCC HepG2 cell line by inducing apoptosis through the intrinsic pathway in a dose-dependent manner, while it displayed non-toxic activity in normal hepatic THLE-2 cells. The selective cytotoxicity of *S. persica* at increasing concentrations in the cancerous HepG2 cell line can be employed for drug development in HCC treatment, aiming for effective inhibition of uncontrolled proliferation and metastasis. The selectivity of the *S. persica* extract towards cancer cells, specifically HepG2, at higher concentrations may be utilized as a therapeutic potential in the treatment of HCC, with no harmful effects on normal healthy cells, thereby increasing the survival rate of patients during HCC treatment.
